# The effect of apparent Police power at demonstrations against right‐wing populism on Protestors' resistance using a virtual reality experiment

**DOI:** 10.1111/bjso.12809

**Published:** 2024-10-07

**Authors:** Julia C. Becker, Lea Hartwich, Helena R. M. Radke

**Affiliations:** ^1^ University of Osnabrueck Osnabrück Germany; ^2^ James Cook University Townsville Queensland Australia

**Keywords:** collective action, legitimacy, police, power, resistance, virtual reality

## Abstract

Based on the Elaborated Social Identity Model of Crowd Behaviour, we tested in two experiments whether a forceful display of police power increases perceptions of illegitimacy of the police and the formation of resistance among protestors. In the high power condition, the police were dressed in riot gear (with helmets, armed with shields and batons). In the low power condition, the police were dressed in regular uniforms. In both studies, people participated in a demonstration against right‐wing populism using a virtual reality setting and were either stopped by the police in riot gear or by the police in regular uniforms. The results of Study 1 (*N* = 155) show that the police in riot gear were evaluated as more illegitimate compared to the police in normal clothing. The results of Study 2 (*N* = 97) replicated this finding and illustrated that police in riot gear (compared to regular uniforms) increased protestors' intentions to engage in direct resistance against the police. This effect was mediated by perceptions of illegitimacy and anger directed at the police. Furthermore, weakly identified protestors were particularly affected by the display of power and were more likely to engage in anti‐police resistance and collective action. Implications are discussed.

## INTRODUCTION

Almost every day we see demonstrations in which protestors are confronted with the police as the executive of state power. We know that police behaviour can contribute to stronger resistance among protestors–but can a change in visibility of police power via variations of police attire be one underlying cause that prompt perceptions of illegitimacy and encourage resistance among activists? In this paper, we examined the influence displays of legal police power has on the formation of resistance. Specifically, we focused on the police as agents of state power and examined whether a manipulation of police attire as an indicator of power affects perceptions of illegitimacy and resistance among protestors in a virtual reality experiment.

## DYNAMIC INTERGROUP PROCESSES CAN SHAPE PERCEPTIONS OF LEGITIMACY AND COLLECTIVE RESISTANCE

There has been a long debate regarding the question of how systems of inequality can survive without eliciting resistance (Jost & Banaji, 1994). It has been argued that sheer force is not helpful to manage unequal relationships (Gramsci, 1971; Jackman, 1994, 2005), but in order to win the voluntary acceptance of inequality consent from the majority of the population needs to be established (Gramsci, 1971). In line with this, Procedural Justice Theory states that people's acceptance of the decisions of legal authorities, legal rules, and their endorsement of the legal system is linked to their assessments of the fairness of the procedures and social processes through which decisions are made by authorities (Tyler & Allan Lind, 2002). Specifically, people rely on how they are treated when they form perceptions and judgements about authorities and the institutions they represent (Maguire et al., 2023). Procedural justice has been shown to increase people's willingness to obey, comply and cooperate with agents of the law and legal authorities (Tyler, 2003, 2011; Tyler & Fagan, 2008), whereas perceptions of procedural injustice trigger affective responses such as anger (Maguire et al., 2023; Scrase, 2021).

In the current research, we focus on perceptions of legitimacy at the *intergroup level*, which are strongly affected by social identity processes. Work on the Elaborated Social Identity Model of Crowd Behaviour (ESIM; Drury & Reicher, 1999, 2000, Reicher, 1996, 1997) suggests that in intergroup situations, people do not mutate into brainless and irrational actors as Le Bon (1947) suggested, but behave in line with ingroup norms. These norms are shaped by a shared social identity that provides guiding principles for appropriate group‐based behaviour. These processes are dynamic, meaning that the intergroup context and ingroup norms are reconceptualized when an outgroup enters the stage: According to self‐categorization processes (Turner et al., 1994), a change of the context (e.g. outgroup behaviour), leads to changes in the self‐categorization of protestors and the emergence of new ingroup norms guide identity‐consonant ingroup actions (Turner et al., 1994). Thus, group actions are at least partially determined by another group's actions.

For instance, Drury and Reicher (2000) use the example of when the police perceive the crowd to be dangerous, they respond by employing riot shields. These riot shields, in turn, can be perceived as illegitimate by protestors and reshape the protestors' ingroup norms and behaviours, resulting in more resistance and confrontational collective action (Stott & Drury, 2000). This has been observed in a seminal study of the ESIM (Reicher, 1996): Protestors who self‐identified as peaceful students exercising their democratic right to protest initially distanced themselves from more radical group members. However, when they were subsequently confronted with police behaviour that they perceived to be illegitimate, they shifted towards supporting confrontational action to resist police violence (see also Drury & Reicher, 2018; Ferris et al., 2019).

It is important to note that these intergroup processes do not emerge in a political vacuum, but these perceptions are biased by police perceptions of the social group that is protesting. It is, for instance, much more likely that the police respond with violence when the group consists of Black people (GBD 2021 Police Violence US Subnational Collaborators), or ethnic minorities (Reinka & Leach, 2017) than when the group consists of whites. In sum, the perceived legitimacy of representatives of state power and the normativity of violent actions changes over time as a response to the actions of an outgroup. Following this, we focus on one specific aspect of police behaviour that is relevant for the present research, how police are dressed, and examine how police uniforms are related to perceptions of power and legitimacy.

## HOW POWER IS EXPRESSED VIA POLICE UNIFORMS

We use the definition of power as the relative control over another's valued outcomes from Fiske and Berdahl (2007). The police as the executive authority have legal state power. Depending on how they are dressed and how they behave, they can either appear as friendly neighbourhood cops or state agents using violent force. These perceptions are affected by observers' attitudes towards the police in general (Jenkins et al., 2021). While police uniforms in general convey power and authority (e.g. Johnson, 2001), militarized police uniform (tactical gear and helmets) can be perceived as an act of symbolic violence (Paul & Birzer, 2004). Symbolic violence inspires fear, subservience and reflects power (Bourdieu, 1977). The militarized ‘riot gear’ uniforms are used to maintain an internal legitimacy by enhancing the role of the police as enforcers of public violence and to symbolically construct a hierarchy between the police and the public (Paul & Birzer, 2004).

Research on the tactical vs. militaristic appearance of police officers in the UK suggests that highly visible weaponry is perceived as threatening but also as a professional representation of authority (Cooke, 2004, see Jenkins et al., 2021). In a study in Canada, it has been found that the police carrying rifles lead participants to indicate that they would be less likely to resist arrest and believed that the officer would be more likely to use excessive force (Blaskovits et al., 2022). Other studies suggest, however, that perceptions of the police as being legitimate (or not) have a stronger influence on participants' ratings of police officers than the manipulations of officer appearance (Jenkins et al., 2021; see also Sunshine & Tyler, 2003). Applied to the present work, when the police are dressed in regular uniforms, they have the relative power over the protestors to stop them from protesting. However, if the police are dressed in militaristic riot gear, they make their power more visible.

In sum, police uniforms represent state power–even more when police officers are dressed in militaristic riot gear. One can ask why police officers dress in riot gear at all. One answer is that they are likely to use riot gear in situations they perceive to be more dangerous in order to maintain law and order, suppress conflict and emphasize the hierarchy (Paul & Birzer, 2004). However, based on the ideas and theories outlined above, a subtle display of power is more likely to win the acceptance of people than a forceful display of that same power (Jackman, 1994). To the extent that protestors see their particular action as legitimate, forceful displays by the police in relation to that action are seen as illegitimate because they are considered disproportionate. Indeed, Stott and colleagues illustrated in the context of football, that crowd conflicts need to be understood as interactions between the crowd and out‐groups such as the police (Drury et al., 2003). They demonstrated that if the police perceive the crowd to be dangerous and treat it as a homogeneous whole, using forceful tactics, protestors perceive the police behaviour to be disproportionate, leading them to unite and legitimize retaliation against the police (Stott & Reicher, 1998). On the other hand, when the police moved away from overt displays of their capability to use force and used a ‘dialogue‐based’ approach, a sense of police legitimacy among football fans emerged leading them to ‘self‐regulate’ in situations of potential intergroup conflict (Stott et al., 2012, see also Stott et al., 2018).

Whereas prior work on the ESIM has been observational in nature, our research set out to test this hypothesis experimentally. Given that it is difficult to ask the police at real life demonstrations to dress a certain way and to observe subsequent effects on the protestors, we used a virtual reality paradigm. Research illustrates that being immersed in virtual reality can elicit similar perceptions and emotions as being in the same situation in real life (e.g. Chirico & Gaggioli, 2019; Dozio et al., 2021). Given that most other work in the context of collective action uses either observations of the protests, memories of past protests or vignette studies, the use of virtual reality is a novel technique in which we can assess emotions and attitudes participants feel directly after their participation.

## PRESENT RESEARCH

We aimed to examine the influence of displays of legal forms of power on the formation of resistance. Specifically, we focused on the police as agents of state power and examined whether a more forceful display of that power increases perceptions of illegitimacy and resistance among protestors. We conducted two experiments in which people participated in a non‐violent demonstration against right‐wing populism in virtual reality and were stopped by the police. We selected the topic of right‐wing populism because, at the time of data collection, demonstrations against right‐wing populism were common, covered positively in the media and supported by leading politicians (e.g. The Guardian, 2024).

In both studies, we included several variables to assess perceptions of and emotions directed at the police (in line with our pre‐registrations), but we mainly focus on the effects of police appearance on perceived legitimacy (Study 1) and direct resistance against the police (Study 1 and 2). Based on the ESIM, we predicted that the police in riot gear (high power condition) compared to regular uniforms (low power condition) would be perceived as less legitimate, less positive (assessed via warmth and competence), more threatening, and would elicit more anger and anxiety among protestors. Furthermore, we predicted that when confronted with the police in riot gear (compared to regular uniforms) protestors would be more likely to engage in direct anti‐police resistance. As an ancillary research question, we also tested whether the protesters become more committed to the campaign aims after exposure to the police at a demonstration. Specifically, protesters could think that the police in riot gear are neither on their side nor neutral, but instead supporting the right‐wing populists, which might motivate them to engage in further protests. Similarly, it is possible that participants may be more willing to protest in the future when the police threatened their right to protest in disproportionate ways.

Finally, we tested whether identification with the protest movement moderates the effects on anti‐police resistance and collective action, because prior work has shown that stronger identification promotes ingroup‐serving behaviour (e.g. Bradford et al., 2014; Spears, 2011). We pre‐registered that those who are highly identified with the protest movement may be more affected by seeing the police in riot gear and more likely to resist. However, based on the ESIM, it is also conceivable that people who do not feel as being part of the wider crowd at the beginning (low identifiers) change towards a more inclusive self‐category after being exposed to the police in riot gear and perceive the police as illegitimate compared to those who see the police in regular uniforms. We started the research with a small pilot study.

## STUDY 1

In a pilot study (which was part of a larger study on a different topic), we assessed with two items whether the police in riot gear is perceived as more powerful than the police in regular uniform. The sample of this pilot study included *N* = 964 participants, 49,1% were female. We used the two items ‘Police in riot gear (with helmets, shields and batons) have more power than police in regular police uniform’ and ‘Police in riot gear are more likely to achieve their goals than police in regular uniform’. Responses were made on seven‐point rating scales (1 = *do not agree at all*, 7 = *agree completely*). We tested these two items against the neutral midpoint (4). The results show that participants thought the police in riot gear has more power, because their agreement with both items was significantly different from the scale midpoint (*M* = 4.80, *SD* = 1.88 and *M* = 5.20, *SD* = 1.68), *t*(963) = 13.12, *p* < .001, 95% CI [.36, .49], *t*(963) = 22.19, *p* < .001, 95% CI [.64, .79], respectively.

### Method

#### Participants

The sample size was determined a priori using the Monte Carlo power analysis for indirect effects (Schoemann et al., 2017; correlations among variables = .30, power = .80, *α* = .05). This indicated that we needed 165 participants to detect a mediation. The study was completed by *N* = 163 participants. Of the 163, one did not complete the resistance measure and seven participants were identified as multivariate outliers (detected via the Mahalanobis distance). These people either agreed or disagreed with two contrary items (‘I am against right‐wing populism’ and ‘right‐wing populism is legitimate’) on a seven‐point rating scale (1 = *do not agree at all*, 7 = *agree completely*). The other participants rejected right‐wing populism (*M* = 6.85, *SD* = .41) and did not think that right‐wing populism is legitimate (*M* = 1.45, *SD* = .81). Most of the final sample of 155 participants were Germans (96%), 74% were female. They were 18–35 years old (*M* = 23.05, *SD* = 3.13). Participants received course credit or were paid 10 € for their participation in the study.

### Procedure

Participants were welcomed to the virtual reality lab by the experimenter and guided to a desk. They first completed the consent form and were instructed that they would be part of a demonstration against right‐wing populism in virtual reality. They were asked to imagine that there was a racist attack against a migrant in the city a few days ago and that today there was a demonstration announced by right‐wing populists in the city centre. In addition, they were told that there was a counter‐protest announced *against* right‐wing populism, which was the demonstration they would attend.

They were instructed to walk together with the other protestors at the speed of the demonstration. They were asked to walk somewhere between the centre and the first third of the demonstration in order to be able to see what was happening at the front of the crowd. If the demonstration stopped, they were instructed to walk to the front and to look around for about 10 seconds.

**FIGURE 1:**
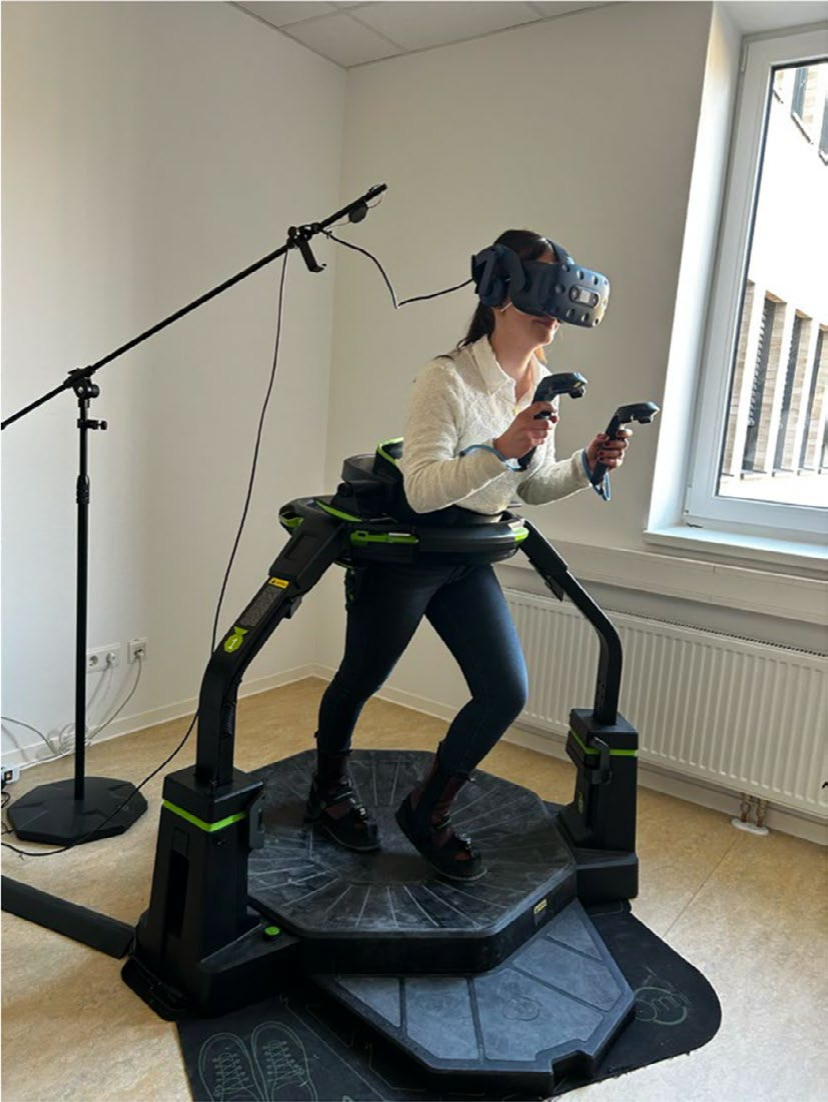
Participant on virtuix omni.

After receiving these instructions, participants put on overshoes and walked into the Virtuix omni. The Virtuix omni is comparable to a treadmill in which participants walk while wearing virtual reality glasses (see Figure 1).

**FIGURE 2:**
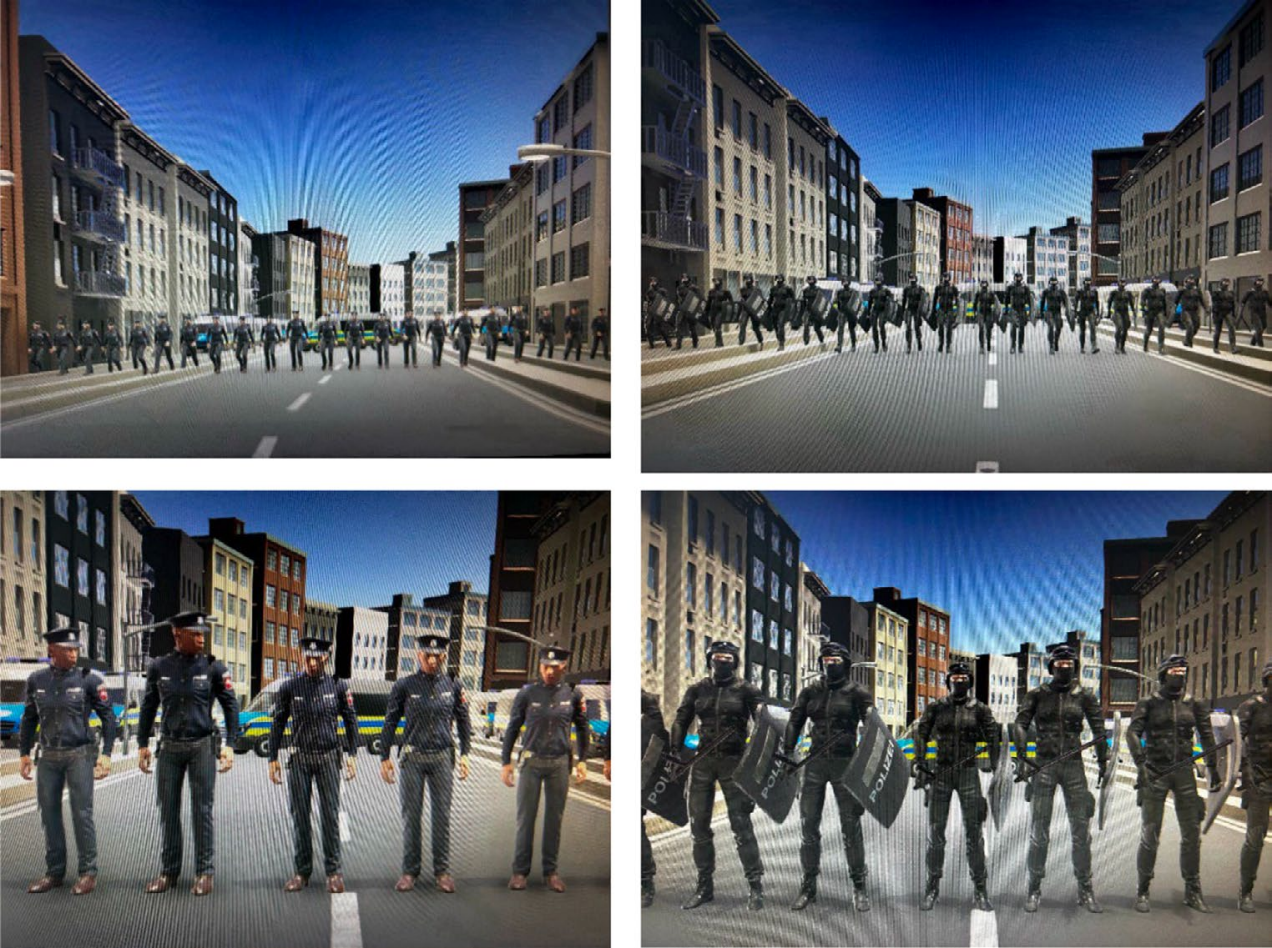
Police chain. Police in regular uniform (left column), police in riot gear (right column).

First, participants learned with a one‐minute video tutorial how to walk on the Virtuix omni. Then, the main application software started and they became part of a demonstration and were asked to walk in the middle of the demonstration (with approximately 150 other protestors). Via headphones, they were exposed to typical noises that happen during a demonstration such as whistles. The protestors walked for approximately 3.5 min through a street in a city with skyscrapers until the police came, created a police blockade and stopped the protest march (Figure 2).

In the low power condition, participants saw the police dressed in regular police uniforms (Figure 2a), in the high power condition, participants saw the police dressed in riot gear with helmets, armed with shields and batons (Figure 2b). These weapons clearly signal that the police have the legal power to get their way by potentially using violence against unarmed protestors. Participants were asked to look at the police for 10 s and to stop the virtual reality experience, go back to their desk and complete a questionnaire with the dependent measures.

This study was pre‐registered at: https://aspredicted.org/GBG_2J3. All data and material are available here: https://osf.io/73gs2/.

### Measures

If not otherwise indicated, responses were made on seven‐point rating scales (1 = *do not agree at all*, 7 = *agree completely*).

***Perceived legitimacy of the behaviour of the police*** was assessed using six self‐developed items (e.g. ‘The behavior of the police was justified’). *α* = .91.

***Negative emotions towards the police*** were assessed using six items. A factor analysis illustrated that feelings of anger (rage, anger, contempt, hate) could be separated from feelings of anxiety (anxiety, fear, Eigenvalues 3.34, 1.45, 0.60…). Thus, we created one factor for *anger‐related emotions* (factor loadings >.79, *α* = .87) and one factor for *anxiety* (factor loadings >.92. *r* = .89).

***Attitudes towards the police (warmth, competence, threat)*** were assessed with four items assessing warmth (The police are nice, likeable, friendly, trustworthy), two items assessing competence (The police are clever, smart) and six items assessing threat perceptions (The police are threatening, assertive, violent, strong, frightening, dangerous). A factor analysis (Eigenvalues 5.63, 2.26, 0.94…) indicated that warmth and competence loaded together on one factor (all factor loadings > .74) and the threat items loaded on a second factor (all factor loadings > .51). Therefore, we created one factor assessing *positive attitudes* towards the police (warmth and competence *α* = .90) and a second factor assessing *threat perceptions*, *α* = .83.

***Anti‐police resistance*** was assessed with two items (e.g. I would have liked to break the police chain, *r* = .89).

***Future collective action intentions*** were assessed with seven items (e.g. In case right‐wing populists demonstrate again: How likely do you think is it that you will participate in a demonstration against right‐wing populism?, *α* = .82).

***Identification with the protest movement*** was assessed with three items (e.g. I identify with the protest movement against right‐wing populism *α* = .87).^1^

### Results

Table 1 presents descriptive statistics and correlations.

**TABLE 1:** Descriptives study 1.

	M (SD) regular uniform	M (SD) riot gear	CA	Resistance	Anger/hate	Anxiety	Pos. Atti‐tudes	Threat	Identity
Legitimacy	4.90 (1.42)	4.02 (1.50)	−.30**	−.43**	−.60**	−.32**	.54**	−.36**	−.08
Collective action (CA)	3.59 (1.22)	3.62 (1.09)		.22**	.34**	.19*	−.10	.12	.53**
Resistance	2.90 (2.07)	2.61 (1.69)			.52**	.22**	−.32**	.19*	.12
Anger/hate	2.15 (1.22)	2.39 (1.35)				.33**	−.48**	.31**	.06
Anxiety	2.88 (1.47)	3.80 (1.86)					−.41**	.62**	.08
Positive attitudes	3.55 (1.17)	3.00 (1.10)						−.47**	.10
Threat	4.22 (.91)	5.14 (.94)							.12
Identity	5.23 (1.26)	4.98 (1.35)							1

**p < .01.

*p < .05.

A MANOVA tested for differences between the two uniform conditions in the seven dependent variables. The overall effect was significant, *F*(7,147) = 8.07, *p* < .001, *η* ^*2*^ = .28. At the univariate level results show that the police dressed in riot gear were perceived to be less legitimate, *F*(1,153) = 13.77, *p* < .001, *η* ^*2*^ = .08, less warm and competent, *F*(1,153) = 8.96, *p* = .003, *η* ^*2*^ = .06, and more threatening, *F*(1,153) = 37.84, *p* < .001, *η* ^*2*^ = .20, compared to the police dressed in regular uniforms. Moreover, participants were more anxious when confronted with the police in riot gear compared to regular uniforms, *F*(1,153) = 11.57, *p* < .001, *η* ^*2*^ = .07. There were no effects of police power on anger, *F*(1,153) = 1.40, *p* = .24, future collective action intentions, *F*(1,153) = .03, *p* = .87, and anti‐police resistance, *F*(1,153) = .90, *p* = .34.

As preregistered, using Process (Hayes, 2018, Model 1, bias‐corrected 95% confidence intervals, 10,000 bootstrap samples) we tested whether identification with the protest movement moderated the effect of police power on future collective action intentions and anti‐police resistance. The interactions were not significant, *b* = −.07, *SE* = .12, *t*(155) = −0.55, *p* = .59, 95% CI [−.31, .17], *b* = −.23, *SE* = .23, *t*(155) = −1.01, *p* = .32, 95% CI [−.69, .23], respectively. Given that we did not find a main effect on anti‐police resistance and future collective action, we did not test for mediation effects. Notably, anger was positively correlated with anti‐police resistance.

### Discussion

As expected, the results show that the police in riot gear were perceived as less legitimate, more threatening, less warm and competent, and elicited stronger feelings of anxiety compared to the police in regular uniforms. This illustrates that a forceful display of state power increases perceptions of threat and illegitimacy. However, the police in riot gear did not affect participant's feeling of anger, anti‐police resistance and future collective action intentions. From our view, it is not surprising that there were no effects on future collective action: attending protest in a different situation in the future is a behaviour that is far away from the direct conflict situation with the police participants were presented with in this study. However, it is more difficult to explain why the police appearance did not motivate resistance in the immediate situation and why participants did not feel more group‐based anger given that the police in riot gear was perceived as less legitimate. We have several suggestions to explain this lack of effect. First, participants were instructed at the beginning of the study that there would be a demonstration of right‐wing populists in the city. Thus, if the police had not stopped the demonstration, they might have clashed with the right‐wing populists. This might have attenuated their anger about being stopped by the police and their intention to break through the police chain to avoid the risk of being exposed to right‐wing populists. Therefore, in Study 2, we focused on the racist attack as a reason for the protest against right‐wing populism and did not mention any further demonstration of right‐wing populists. Second, we realized that the items assessing anti‐police resistance were not optimal, as they pointed in the direction of non‐normative collective action and to direct contact with the police (breaking through the police chain). We saw that protestors felt more anxious and threatened by the police in riot gear. Thus, they might want to avoid direct physical contact with the armed police. Therefore, we aimed to improve the measure of anti‐police resistance in Study 2 by broadening this concept and including different activities that do not require a physical confrontation with the police. Third, the anti‐police resistance measure was framed in the past ‘I would have liked to break the police chain’–in Study 2, we asked participants to re‐imagine the situation they had experienced in the virtual reality in their minds and to evaluate how they would like to act in this immediate situation.

## STUDY 2

The goal of Study 2 was to replicate that higher power police (in riot gear) increased perceptions of illegitimacy and negative emotions compared to lower power police (in regular uniforms) using the same virtual reality paradigm. We also improved the scale assessing anti‐police resistance. Furthermore, we measured identification with the protest movement before and after the manipulation for a more appropriate test of T1 identification as a moderator. Finally, we aimed to test the serial mediation whether police uniform affects anti‐police resistance because protestors perceive the police to be less legitimate and respond with increased anger. This study was pre‐registered: https://aspredicted.org/38C_VL9. All data and material is available here: https://osf.io/73gs2/.

### Method

#### Design

The design was similar to the design of Study 1 except with a few changes. First, we introduced a running condition in a 2 (police uniform: normal police clothing vs. riot gear) × 2 (speed: walking vs. running) condition. We added the running condition, because we reasoned that participants doing this sport activity together and being physically aroused might feel more united with the other protesters and accordingly feel more anger and have stronger perceptions of illegitimacy compared to those in the walking condition. However, data of the running condition could not be used due to technical problems that occurred in this condition. The most prevalent problem was that people were not able to keep up with the speed of the other protestors, fell behind, ended the study behind the demonstration and could not see the police. Thus, we used the data from the ‘walking condition’ only. Second, participants were provided with the information that there was a racist attack against a migrant as the only reason for the demonstration against right‐wing populism (we did not say that there was a demonstration of right‐wing populists in the city as well). Third, we assessed T1 identification before the manipulation, directly after providing informed consent and T2 identification after the VR experience. Fourth, we asked participants when completing the questionnaire to re‐imagine the situation they experienced 1–2 min ago, when they were immersed in the VR simulation and to report their direct action intentions (instead of asking how they would have liked to behave).

#### Participants

The original power analysis was based on a 2 × 2 analysis (see https://aspredicted.org/38C_VL9). However, as noted above, we could not use the data from the ‘running condition’. A power analysis for the one‐factorial design (g*power, test of mean differences, *d* = .5, power = .80, *α* = .05, one‐tailed) indicated that we needed 102 participants. Data (*N* = 102) were collected in the same VR lab as in Study 1. Of the 102 people who completed the survey, one did not complete the collective action scales. Four further participants were multivariate outliers (detected with the same measures as in Study 1). The remaining participants indicated to be against right‐wing populism (*M* = 6.66, *SD* = .73) and did not think that right‐wing populism was legitimate (*M* = 1.58, *SD* = .86). Most of the final sample of 97 participants were Germans (88%, 6% came from other European countries, 5% from Asia, 1% from South America), 63% were female. They were 18–68 years old (*M* = 24.57, *SD* = 6.36). Participants received course credit or were paid with 10 € for their participation in the study.

### Measures

***Perceived legitimacy of the police*** was assessed with the same items as in Study 1, *α* = .87.

***Negative emotions towards the police*** were assessed with the same items as in Study 1. Again, a factor analysis (Eigenvalues 2.83, 1.78, 0.83…) illustrated that anger‐related emotions (all factor loadings >.73) could be separated from anxiety‐related emotions (all factor loadings >.96). Thus, we created one factor for anger‐related emotions (*α* = .84) and one factor for anxiety, *r* = .89.

***Anti‐police resistance*** was assessed with nine items (e.g. ‘I would like to continue the protest and confuse the police with minor actions’, ‘I would like to chant with others that the police should go away’, ‘I would like to sign a petition against the police behavior’). One item was dropped from the scale (‘I would like to show the police that we cannot be stopped and participate in a sit‐in’), because of a low item‐total correlation (<.30). The final scale was reliable (*α* = .85).

**Future collective action intentions** were assessed with the same items as in Study 1 (*α* = .75).

***Identification with the protest movement*** was assessed before and after the VR experience with the same items as in Study 1 (T1 *α* = .83, T2 *α* = .89).

***Moral convictions*** 2 were assessed with five items (e.g. ‘it is our moral duty to fight against right‐wing populism’, ‘it is ok to break a law when one is morally right’). The five items created a reliable scale (*α* = .71).

### Results and discussion

Table 2 presents descriptive statistics and correlations.

**TABLE 2:** Descriptives study 2.

	M (SD) regular uniform	M (SD) riot gear	CA	Resistance	Anger/hate	Anxiety	Identity T1	Identity T2	Moral convict‐ions
Legitimacy	3.89 (1.36)	2.57 (1.09)	−.23*	−.46**	−.47**	−.16	−.26**	−.29**	−.29**
Collective action (CA)	3.20 (1.20)	3.29 (.81)		.53**	.47**	−.01	.56**	.47**	.58**
Resistance	3.60 (1.57)	4.26 (1.09)			.67**	.02	.42**	.49**	.52**
Anger/hate	2.66 (1.34)	3.22 (1.32)				.14	.41**	.58**	.39**
Anxiety	2.87 (1.66)	3.73 (1.68)					−.12	.04	−.03
Identity T1	5.47 (1.24)	5.31 (1.18)						.66**	.50**
Identity T2	4.84 (1.57)	4.95 (1.27)							.49**
Moral convictions	4.38 (2.00)	4.17 (.98)							1

**p < .01.

*p < .05.

We used a MANOVA to test for differences between the police uniform conditions on perceived legitimacy of the police, anger towards the police, anxiety because of the police, moral convictions, anti‐police resistance and future collective action as the dependent measures, *F*(6,90) = 7.95, *p* < .001, *η* ^*2*^ = .35. As expected, at the univariate level, participants in the riot gear condition perceived the police as less legitimate, *F*(1,95) = 28.10, *p* < .001, *η* ^*2*^ = .23, were angrier, *F*(1,95) = 4.35, *p* = .04, *η* ^*2*^ = .04, and more anxious, *F*(1,95) = 6.51, *p* = .01, *η* ^*2*^ = .06, and were more interested in anti‐police resistance compared to those in the regular uniform condition, *F*(1,95) = 5.93, *p* = .017, *η* ^*2*^ = .06. However, there were no effect of condition on moral convictions, *F*(1,95) = 0.9, *p* = .35, *η* ^*2*^ = .009, and future collective action intentions, *F*(1,95) = 0.2, *p* = .65, *η* ^*2*^ = .002.

**FIGURE 3:**
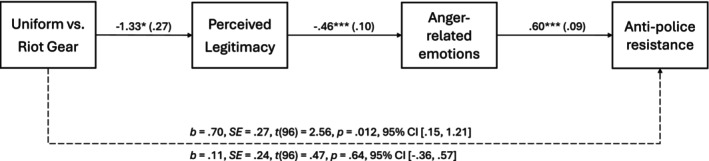
Serial Mediation Study 2. ***p < .001, **p < .01, *p < .05, total effect above the dashed line, direct effect below the dashed line.

To test whether the presence of police in riot gear leads to stronger intentions for anti‐police resistance, because it increases perceptions of illegitimacy, which in turn, increases anger directed at the police, we conducted a mediation analysis using PROCESS (Hayes, 2018, Model 6, bias‐corrected 95% confidence intervals, 10,000 bootstrap samples) with police uniform (normal clothing vs. riot gear) as predictor, anti‐police resistance as the outcome, and perceptions of legitimacy and anger as serial mediators. The total effect of police uniform on anti‐police resistance was significant, *b* = .70, *SE* = .27, *t*(96) = 2.56, *p* = .012, 95% CI [.15, 1.21]. Police uniform was related to perceptions of legitimacy, *b* = −1.33, *SE* = .25, *t*(96) = −5.34, *p* < .001, 95% CI [−1.82, −.83]. Legitimacy perceptions were related to anger, *b* = −.46, *SE* = .10, *t*(96) = −4.58, *p* < .001, 95% CI [−.66, −.26], and anger, in turn, to anti‐police resistance, *b* = .60, *SE* = .09, *t*(96) = 6.89, *p* < .001, 95% CI [.42, .77]. When all variables were entered into the model, the total effect of police power on anti‐police resistance was reduced to a non‐significant direct effect, *b* = .11, *SE* = .24, *t*(96) = .47, *p* = .64, 95% CI [−.36, .57]. As expected, the effect of police power on anti‐police resistance was mediated via perceptions of legitimacy and anger‐related emotions, *B* = .36, *SE* = .11, 95% CI [.16, .60, see Figure 3].

**FIGURE 4:**
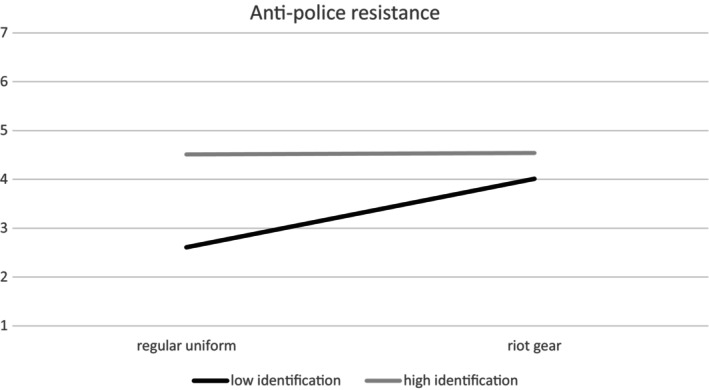
Simple slope analysis: Relation between identification and anti‐police resistance depending on police uniform.

We preregistered to test whether the effects of police uniform on anti‐police resistance and future collective action are moderated by identification with the protest movement at T1. We conducted an analysis with Process Model 1. T1 identification moderated the effects of police uniform on anti‐police resistance (*b* = −.57, *SE* = .20, *t*(96) = −2.88, *p* = .005, 95% CI [−.96, −.18]). Figure 4 illustrates these findings: the weakly identified (1 *SD* below the mean) in the riot gear condition were more interested in anti‐police resistance compared to the weakly identified in the regular uniform condition, *b* = 1.38, *SE* = .33, *t*(96) = 4.12, *p* < .001, 95% CI [.72, 2.04], whereas there was no difference between conditions for the highly identified with the protest movement (1 *SD* above the mean), *b* = .02, *SE* = .33, *t*(96) = 0.06, *p* = .95, 95% CI [−.64, .68].

**FIGURE 5:**
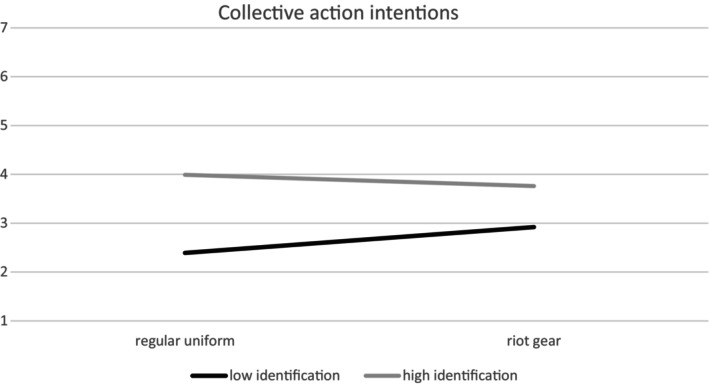
Simple slope analysis: Relation between identification and collective action intentions depending on police uniform.

Similarly, T1 identification moderated the effect of police power on future collective action intentions, (*b* = −.31, *SE* = .14, *t*(96) = −2.16, *p* = .03, 95% CI [−.59, −.03]). Figure 5 illustrates that the weakly identified (1 *SD* below the mean) in the riot gear condition were more interested in collective action compared to the weakly identified in the regular uniform condition, *b* = .53, *SE* = .24, *t*(96) = 2.21, *p* = .03, 95% CI [.05, 1.01], whereas there was no difference between conditions for the highly identified (1 *SD* above the mean), *b* = −.20, *SE* = .24, *t*(96) = −0.85, *p* = .40, 95% CI [−.68, .27].

We explored whether T1 identification would moderate the effect of police power on perceived legitimacy, which was not the case (*b* = .30, *SE* = .20, *t*(96) = 1.50, *p* = .14, 95% CI [−.10, .69]).

For reasons of completeness, we tested whether the effect of police power on anti‐police resistance/collective action intentions mediated by perceptions of illegitimacy and anger was moderated by T1 identification (using PROCESS, model 8). The index for moderated mediation was not significant, *b* = −.12, *SE* = .10, 95% CI [−.34, .07]/*b* = −.05, *SE* = .05, 95% CI [−.17, .02], respectively.

We conclude that police in riot gear can elicit direct anti‐police resistance, because protestors perceive the police in riot gear as less legitimate and respond with more anger. Moreover, we also found first evidence that police in riot gear (compared to regular uniforms) strengthens intentions for collective action outside of the immediate protest context among those weakly identified, whereas the highly identified were more interested in collective action in both conditions. This finding is in line with the ESIM. We discuss the implications of our results below.

## GENERAL DISCUSSION

The results from two studies using a virtual reality paradigm demonstrated that when the police make their power clearly visible by dressing in riot gear, protestors perceive the police's appearance and behaviour as less legitimate (Study 1 and 2) and are more willing to engage in direct anti‐police resistance (Study 2) compared to a condition in which the police power is less visible by dressing in regular police uniforms. Thus, the present research contributes to our understanding of how a more forceful display of legal forms of power by the authority does not perpetuate the status quo, but, in contrast, leads protestors to perceive state power as less legitimate and shape a dynamic interaction between social groups by motivating protestors to build oppositional power and to participate in collective resistance. This work adds to the collective action literature by showing that protestors' actions are a dynamic function of the way protesters are treated at crowd events. In a nutshell, we find that for the same police action (preventing a protest march moving forward), seeing the police in riot gear adds a significantly higher sense of being treated unfairly, which in turn had consequences for action intentions.

Interestingly, in Study 2, we found an interaction effect between the police uniform condition and Time 1 movement identification on anti‐police resistance. Participants more weakly identified with the protest movement at the beginning of the study demonstrated more interest in anti‐police resistance when they were confronted with the police in riot gear compared to regular uniforms. This finding nicely fits to observational studies conducted to test the ESIM, in which Drury and Reicher (2018) observed that those who were initially somewhat sceptically about the more radical people within the movement changed their views when the police reacted in an illegitimate way. Further, it is likely that those who did not strongly feel part of the movement in the beginning became more committed to the theme of the protest (anti‐right‐wing populism) after their experiences with the police in riot gear at the demonstration. However, due to the small sample size, the moderation should be interpreted with caution and should be replicated in future work using a larger sample size.

The present work has several implications. It contributes to the growing body of work showing that protestors do not act unpredictable and arbitrary based on their instincts, but that protestors' actions are strategic and chosen for a reason. Our findings show that increased interest in resistance reflects the rational acting out of the defining dimensions of the protestor's collective identity in relation to the police appearance (see also Stott & Drury, 2000). However, we do not know whether the protestors who are confronted with very visible police power intend to act against the police because they feel unjustly deprived of their right to protest in this particular situation, or whether this experience (when it happens in real life) has more long‐term implications for individuals and might start to radicalize protestors. Related to this, it is important to keep in mind that the police do not perceive all crowds to be similarly dangerous and that in real life some groups being more likely to be confronted with police in riot gear than other groups. Black people and ethnic minorities are more likely to experience violence than white people (GBD 2021 Police Violence US Subnational Collaborators, 2021; Reinka & Leach, 2017). This is also what research on the ‘Police Officer's Dilemma’ suggests. Black people and ethnic minorities such as Arab Muslims are more likely to be shot than white targets (Correll et al., 2002; Essien et al., 2017). The idea of the velvet glove (Gramsci, 1971; Jackman, 1994) suggests that systems of inequality survive for centuries when powerholders can establish the voluntary acceptance of inequality from the majority without using force. Our findings support the idea that a forceful display of power leads to interest in resistance and non‐compliance.

### Limitations and directions for future research

There are some limitations within these studies. Although perceptions and emotions can be similar in the analogue and virtual reality (e.g. Chirico & Gaggioli, 2019; Dozio et al., 2021), we would argue that effects experienced in the analogue reality might be even stronger than those observed in the virtual reality. On the one hand, it is possible that the police in riot gear leads to stronger feelings of threat and anxiety in the analogue reality and might rather inhibit than encourage intentions to resist. On the other hand, being stopped by the police in the analogue reality might elicit stronger feelings of anger and perceptions of illegitimacy (than in the virtual reality) which might motivate stronger anti‐police resistance. Nevertheless, we do note that our findings go beyond the previous literature by (1) providing an experimental test of ESIM which is limited in the current literature and (2) going beyond vignette studies but still maintaining rigorous experimental control using a virtual reality paradigm.

Second, it could be argued that the results can be explained by the weapon effect. The weapon effect suggests that the mere presence of weapons can increase aggression (Bushman & Romer, 2021). Thus, it is possible that the protestors responded to the batons/riot sticks by becoming more aggressive and this increased aggression triggered other emotional reactions (anger and fear) and anti‐police resistance. However, first, a meta‐analytic review illustrated that the weapon effect might be overestimated (Benjamin et al., 2018). Second, we believe that it is more likely that it was the perception of illegitimacy that triggered the effects given that results of Study 2 provided evidence for a moderation, which also qualified the effects: weakly identified were more likely to increase their intention to protest and it is unlikely that only the weakly identified became more aggressive. However, future research could test whether the riot gear with helmets and armour is enough to elicit these effects or whether shields and batons/riot sticks need to be observable as well.

Third, when comparing the means between the two studies, it is salient that the means of legitimacy perceptions were around the midpoint of the scale in Study 1 and significantly lower in Study 2. We believe this goes back to the underlying reason for the protest, which differed in Study 1 and Study 2. In Study 1, participants believed that there would be a protest by right‐wing populists. Thus, they might have thought that it was legitimate to be stopped by the police in order to avoid contact with the right‐wing populists. In Study 2, we did not provide any information that there was a protest by right‐wing populists but informed participants that the reason for their protest was a racist attack some days before. It is likely that the composition of our sample contributed to the relatively moderate perceptions of legitimacy. Our studies were conducted with mostly white participants who were surrounded by mostly white people at the demonstration in virtual reality. Furthermore, more women than men participated in the studies, but women are less likely to be targeted by police controls than men (SVR‐migration, 2023). Thus, it is likely that that most of our highly educated white female participants are not confronted with the police in their daily lives. Thus, it would be interesting to replicate the study with people who are more often confronted with the police, for instance, by experiencing individual or structural racism (e.g. Trawalter et al., 2020). It is likely that people who make more often racist experiences with the police are more likely to perceive their behaviour as illegitimate. Finally, although we included a pilot study that confirmed that the police in riot gear were perceived as being more powerful compared to the police in regular uniforms, we did not ask for perceived power of the police in both studies. This limitation should be addressed in future research.

### Conclusion

Prior work examined effects of police behaviour on protestors' attitudes using behavioural observations or surveys. In the current work, we used a virtual reality paradigm because it combines the experience of participating in a demonstration with the possibility to introduce a standardized manipulation. The results of two experiments suggest that for the same police action (preventing a protest march moving forward), seeing the police in riot gear induces a significantly higher sense of being treated unfairly (in both studies), which in turn predicted participant's willingness to engage in anti‐police resistance (Study 2). In line with the ESIM, particularly weakly identified protestors were affected by the display of power and were more likely to engage in anti‐police resistance and collective action. This research contributes to our understanding of how a more forceful display of legal forms of power by the authority does not perpetuate the status quo, but, in contrast, lead protestors to perceive state power as less legitimate and shape a dynamic interaction between social groups by motivating protestors to build oppositional power and to participate in collective resistance.

## AUTHOR CONTRIBUTIONS

**Julia C. Becker:** Conceptualization; investigation; funding acquisition; writing – original draft; methodology; validation; visualization; software; formal analysis; project administration; data curation; resources. **Lea Hartwich:** Conceptualization; writing – review and editing. **Helena R. M. Radke:** Conceptualization; writing – review and editing.

## CONFLICT OF INTEREST STATEMENT

The authors have no conflict of interests to declare that are relevant to the content of this article.

## Data Availability

Data is available here: https://osf.io/73gs2/

## Footnotes

^1^
We also assessed attitudes towards the protestors, group efficacy, moral convictions, positive emotions for exploratory purposes. The full material is uploaded on OSF.

^2^
We included this variable because we preregistered that moral convictions might be affected by the police appearance. We reasoned that participants feel stronger moral convictions when seeing the police in riot gear. In addition, we also assessed personal emotions, perceptions of the police and protestors and group efficacy. All materials are uploaded on OSF.
